# First Molecularly Confirmed Outbreak of Bovine Pythiosis Caused by *Pythium insidiosum* in the Amazon Biome

**DOI:** 10.3390/pathogens15040409

**Published:** 2026-04-09

**Authors:** Janayna Barroso dos Santos, Hanna Gabriela da Silva Oliveira, André de Medeiros Costa Lins, Edson Moleta Colodel, Agnes de Souza Lima, Henrique dos Anjos Bomjardim, Flavio Roberto Chaves da Silva, Cíntia Daudt, Valeria Dutra, Felipe Masiero Salvarani

**Affiliations:** 1 Instituto de Medicina Veterinária, Universidade Federal do Pará, Castanhal 68740-970, PA, Brazil; janayna.barroso@castanhal.ufpa.br (J.B.d.S.); hanna.oliveira@castanhal.ufpa.br (H.G.d.S.O.); andre.lins@castanhal.ufpa.br (A.d.M.C.L.); 2 Faculdade de Medicina Veterinária, Universidade Federal de Mato Grosso, Cuiabá 78060-900, MT, Brazil; moleta@gmail.com (E.M.C.); valeriadutra.dutra@gmail.com (V.D.); 3 Laboratório de Virologia Geral e Parasitologia, Centro de Ciências Biológicas e da Natureza, Universidade Federal do Acre, Campus Universitário, BR 364, Km 04-Distrito Industrial, Rio Branco 69920-900, AC, Brazil; agnes.lima@ufac.br (A.d.S.L.); flavio.silva@ufac.br (F.R.C.d.S.); cintia.daudt@ufac.br (C.D.); 4 Faculdade de Medicina Veterinária, Universidade do Norte do Tocantins, Araguaína 77826-612, TO, Brazil; henrique.bomjardim@ufnt.edu.br

**Keywords:** oomycete infection, quantitative PCR (qPCR), flood-prone pastures, water-associated pathogens, field outbreak investigation, One Health surveillance

## Abstract

Pythiosis is a neglected infectious disease caused by the aquatic oomycete *Pythium insidiosum* and remains underrecognized in cattle, particularly in tropical regions. Here, we report the first molecularly confirmed outbreak of bovine pythiosis in the Amazon biome, affecting more than 400 animals raised under extensive production systems and areas with prolonged exposure to standing water. Clinically affected cattle presented ulcerative and exudative cutaneous lesions, predominantly involving the distal limbs. Given the diagnostic challenges associated with pythiosis, etiological confirmation was achieved through quantitative PCR (qPCR) targeting the internal transcribed spacer (ITS) region of *P. insidiosum*, providing rapid and specific molecular detection during the outbreak investigation. Therapeutic interventions were implemented as part of routine field management, including intramuscular triamcinolone combined with topical copper sulfate; this regimen was associated with clinical improvement in a substantial proportion of affected animals, though treatment efficacy was not formally evaluated. The outbreak occurred in flood-prone pastures during the rainy season, highlighting the role of aquatic environments in pathogen transmission. These findings expand the current understanding of bovine pythiosis in tropical ecosystems and underscore the importance of molecular diagnostics, outbreak surveillance, and a One Health approach for the identification and management of water-associated pathogens in livestock.

## 1. Introduction

Pythiosis is a neglected infectious disease caused by *P. insidiosum*, an aquatic oomycete endemic to tropical and subtropical regions. Unlike true fungi, *P. insidiosum* produces motile biflagellate zoospores and is strictly associated with stagnant or slow-moving warm water, which explains its strong epidemiological link to flood-prone environments and water-associated transmission cycles [1,2,3,4]. The pathogen infects a wide range of hosts, causing predominantly cutaneous disease in animals and vascular or ocular forms in humans [5]. Although horses and dogs are most frequently affected, bovine pythiosis remains underreported, likely due to variable clinical presentation, spontaneous lesion regression in some outbreaks, and frequent misdiagnosis as other ulcerative dermatoses [6,7,8]. While often reported in tropical and subtropical regions of South America, Southeast Asia, and parts of the southern United States, cases of pythiosis have been increasingly documented worldwide, highlighting its expanding geographical relevance and the need for global awareness [5].

In cattle, pythiosis typically presents as ulcerative cutaneous lesions affecting body regions with prolonged contact with water. Accurate etiological diagnosis is challenging because clinical findings are nonspecific and empirical antifungal approaches are ineffective against oomycetes. Consequently, molecular diagnostic methods, particularly PCR-based assays targeting the internal transcribed spacer (ITS) region, have become essential for definitive identification of *P. insidiosum*, especially in outbreak settings where rapid confirmation is critical [9,10,11,12,13].

Despite increasing recognition of pythiosis worldwide, molecularly confirmed bovine cases remain scarce, and no previous molecularly confirmed outbreaks have been reported in cattle from the Amazon biome. This region is characterized by extensive flood-prone pastures, in the rainy season, high rainfall and temperatures, and ongoing environmental change, conditions that favor the persistence of *P. insidiosum* and other water-associated pathogens [14,15,16,17,18]. In this context, the present study reports the first molecularly confirmed outbreak of bovine pythiosis in the Amazon biome, emphasizing the role of molecular diagnosis in outbreak investigation and One Health surveillance in tropical livestock systems.

## 2. Materials and Methods

### 2.1. Study Area and Epidemiological Background

The outbreak investigation was conducted between January and April 2025 in a commercial beef cattle farm located in Paragominas (02°59′45″ S 47°21′10″ W) the Amazon biome, Pará State, northern Brazil. The affected herd comprised approximately 850 cattle raised under extensive grazing conditions. The outbreak occurred during the peak of the regional rainy season, a period characterized by high precipitation (average accumulated rainfall greater than 1000 mm), sustained ambient temperatures ranging from 30 to 34 °C, and prolonged flooding of pasturelands. Large grazing areas remained waterlogged for several weeks, with extensive formation of stagnant or slow-moving water bodies.

### 2.2. Clinical Assessment and Case Definition

This investigation was designed as an observational field study focused on outbreak documentation and management, conducted under routine farm conditions, and not as a controlled therapeutic trial. The primary objective was to characterize the clinical presentation, epidemiological features, and molecular confirmation of a suspected pythiosis outbreak, as well as to describe the therapeutic approaches applied during outbreak management. Treatment decisions were based on lesion severity and clinical judgment, reflecting standard veterinary practice in field conditions, without randomization, experimental allocation, or predefined comparative endpoints. Therefore, therapeutic outcomes are reported descriptively and should be interpreted as observational field findings rather than evidence of causal efficacy or superiority between protocols, in line with recommendations for outbreak investigations and case-based veterinary studies [6,8,19].

**Figure 1:**
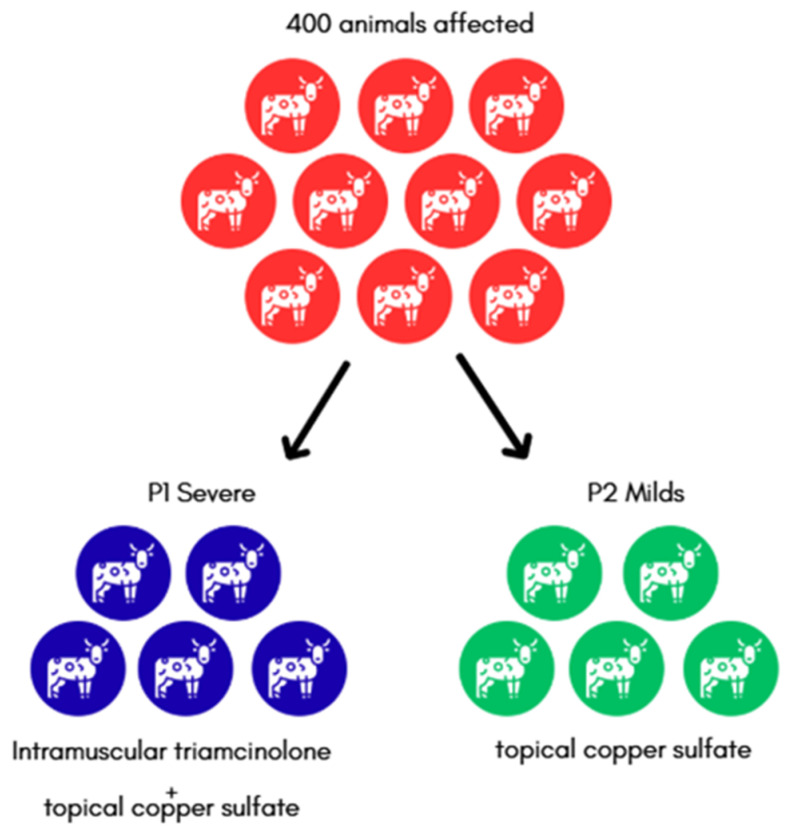
Division of affected cattle into severe (P1) and mild (P2) cases.

All animals underwent systematic clinical examination by licensed veterinarians. Case definition was based on the presence of characteristic cutaneous lesions compatible with pythiosis, including ulcerative, exudative, and necrotic skin lesions with irregular margins. A total of 400 cattle (47.1% of the herd) were identified as clinically affected through direct inspection. Lesions were predominantly located on the distal portions of the limbs, with a higher frequency on the hind limbs, consistent with prolonged exposure to flooded pastures. For descriptive and management purposes, affected animals were stratified into two clinical categories based on lesion number and extent. Animals presenting with multiple or extensive ulcerative lesions were classified as severe cases (P1–213 animals) treated with intramuscular triamcinolone combined with topical copper sulfate, whereas animals presenting a single, small, and localized lesion were classified as mild cases (P2–187 animals) treated only topical copper sulfate (Figure 1). No animals exhibited marked lameness or poor body condition at the time of clinical evaluation.

### 2.3. Sample Collection, Histopathological and Molecular Diagnostic

Given the large number of clinically affected animals during the outbreak, a targeted, representative, and pragmatic sampling strategy was adopted, in accordance with established approaches for field investigations of pythiosis and other environmentally associated oomycete infections [6,19]. The primary objective of sampling was etiological confirmation at the herd level while minimizing animal handling and invasive procedures during an active outbreak scenario.

Cutaneous tissue samples were collected from a subset of cattle presenting typical ulcerative lesions, selected to represent different paddocks, lesion stages, and degrees of clinical severity. This strategy is widely accepted in outbreak investigations, in which concordant clinical, epidemiological, and molecular findings allow robust identification of the causative agent without exhaustive sampling of all affected animals [6]. Fifteen animals presenting active lesions defined as lesions identified shortly before sampling and exhibiting ongoing inflammatory activity were initially selected. To perform the skin biopsy, fragments from the margins of the lesions were collected aseptically and painlessly using local anesthesia (2% lidocaine) under sterile conditions. The tissues were also fixed in 10% neutral buffered formalin and underwent conventional histopathological processing and staining with hematoxylin and eosin (H&E) for general evaluation and with Grocott methenamine silver (GMS) staining for visualization of hyphal structures [7]. For molecular analyses, fresh lesion tissue biopsies from ten bovines were available and processed.

Biopsies were aseptically obtained from the active peripheral margins of cutaneous lesions, where the pathogen burden and the likelihood of detecting viable *P. insidiosum* structures are expected to be highest, in accordance with established recommendations for the molecular diagnosis of pythiosis [4,9]. Fresh lesion tissue biopsies were defined a priori as the standard specimen type to ensure diagnostic accuracy and maximize analytical sensitivity. Collected tissues were preserved in RNAlater™ (Invitrogen, Foster City, CA, USA) and stored at −20 °C until laboratory processing. No lesion swabs or superficial samples were used, thereby maintaining methodological uniformity and minimizing variability associated with surface contamination and low pathogen load.

Collected tissues were divided into two portions: one fixed in formalin for histopathology and the other preserved in RNAlater™ (Invitrogen, Foster City, CA, USA) and stored at −20 °C until DNA extraction. DNA was extracted from fresh tissue samples (*n* = 10) using the DNeasy Blood & Tissue Kit (Qiagen, Hilden, Nordrhein-Westfalen, Germany), according to the manufacturer’s instructions.

Molecular detection of *P. insidiosum* was performed by real-time quantitative PCR (qPCR) targeting the internal transcribed spacer (ITS) region of ribosomal DNA, using species-specific primers (Forward: 5′-TTCCTGCCCTTGGTCATTTAG-3′; Reverse: 5′-GATCTGCGTTCTTCATCGATGC-3′) and hydrolysis probes, as previously validated for high specificity and sensitivity [9,10]. Amplification reactions were conducted on a StepOnePlus™ Real-Time PCR System (Applied Biosystems, Foster City, CA, USA). A cycle threshold (Ct) value ≤ 35 was considered positive. Each run included positive controls (reference *P. insidiosum* DNA) and no-template negative controls to monitor assay performance.

To further strengthen species-level confirmation, ITS region sequencing was performed on a subset of qPCR-positive samples. Amplicons were purified and subjected to bidirectional Sanger sequencing. Consensus sequences were assembled and compared with reference sequences deposited in GenBank using BLASTn (BLAST+ version 2.17.0). Species identification was confirmed when sequence identity exceeded 99% with validated *P. insidiosum* reference strains, in accordance with criteria adopted in previous molecular and phylogenetic studies [6,13,20]. Although sequencing was performed in a limited number of cases, this step provided an additional layer of molecular validation, reinforcing the diagnostic robustness of the outbreak investigation.

### 2.4. Treatment Protocol

Therapeutic management was conducted as part of an observational outbreak response, rather than a controlled therapeutic trial. Two treatment protocols were implemented based on previously published evidence and adapted to field conditions, lesion severity, and animal handling feasibility [17,19]. Animals presenting more extensive or progressive lesions were managed under Protocol 1 (P1), consisting of intramuscular administration of triamcinolone acetonide (Retardo Esteróide^®^, Ceva, Juatuba, Brazil) once weekly for three consecutive weeks, combined with topical application of a 5% copper sulfate to the lesions twice weekly for the same period. Animals with milder or localized lesions were managed under Protocol 2 (P2), which consisted exclusively of daily topical application of a 5% copper sulfate solution until complete lesion closure [20,21,22,23]. The selection of these protocols was guided by the documented immunomodulatory effects of corticosteroids on the exuberant inflammatory response associated with pythiosis, as well as the reported direct cytotoxic activity of copper-based compounds against *P. insidiosum* hyphal elements [4,24]. No causal or comparative inference regarding treatment efficacy was intended, and therapeutic outcomes were recorded descriptively as part of the field outbreak management.

### 2.5. Ethical Considerations

All procedures involving animals were approved by the Institutional Committee for Animal Care and Use of the Federal University of Pará (CEUA-UFPA; protocol no. 6261300323). Animal handling and sample collection were performed in accordance with international guidelines for ethical veterinary research, with efforts made to minimize discomfort, stress, and invasiveness during all procedures.

## 3. Results

### 3.1. Epidemiological Characterization of the Outbreak

**Figure 2:**
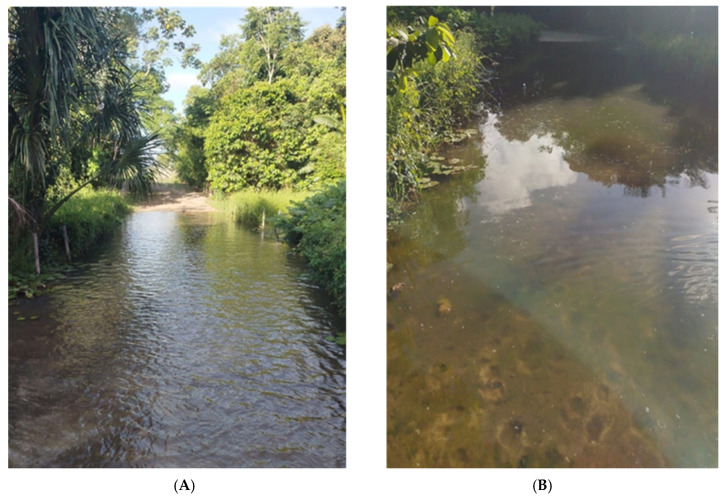
(**A**) Flooded area of stagnant water between pastures, characteristic of the Amazon Biome during rainy season. (**B**) Flooded area of stagnant water with mud and marks of cattle trampling.

Between January and April 2025, a large-scale outbreak of cutaneous pythiosis was identified on a commercial beef cattle farm located in the state of Pará, eastern Amazon biome (02°59′45″ S 47°21′10″ W). The farm maintained a total herd of 850 male cattle managed under an extensive production system with rotational grazing of *Panicum maximum* cv. Mombaça. During the Amazonian rainy season, substantial areas of the property become persistently flooded, and cattle had unrestricted access to these waterlogged paddocks (Figure 2).

Of the 850 animals examined during the outbreak investigation, 400 cattle (47.1%) fulfilled the predefined clinical case definition for suspected cutaneous pythiosis and were included in the descriptive epidemiological analysis. All affected animals were in the rearing phase, predominantly crossbred and Nelore cattle, aged between 8 months and 2.5 years. The overall morbidity rate of the outbreak was therefore 47%. No mortality attributable to pythiosis was recorded during the observation period.

**Figure 3:**
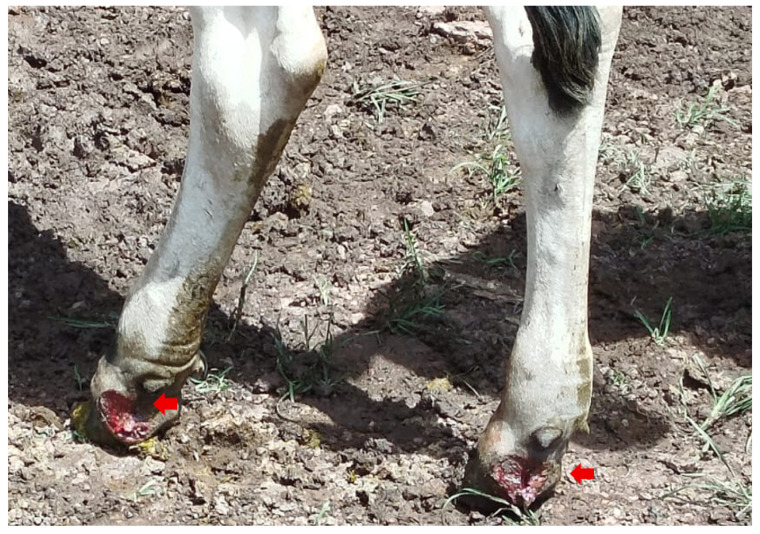
Ulcerated skin lesions, with an alopecic, concave surface, granular appearance and well-defined borders, tending to be circular, measuring 10 cm and 7 cm in size, located on the pastern, lateroplantar region of the left pelvic limb and medioplantar region of the right pelvic limb.

Clinical examination revealed that systemic parameters including heart rate, respiratory rate, rectal temperature, and ruminal motility remained within physiological reference ranges in all affected animals. Notably, none of the cattle exhibited lameness or significant loss of body condition during the course of the outbreak or treatment period, suggesting disease localization to cutaneous tissues without evident systemic involvement (Figure 3).

### 3.2. Clinical Presentation and Lesion Characteristic

**Figure 4:**
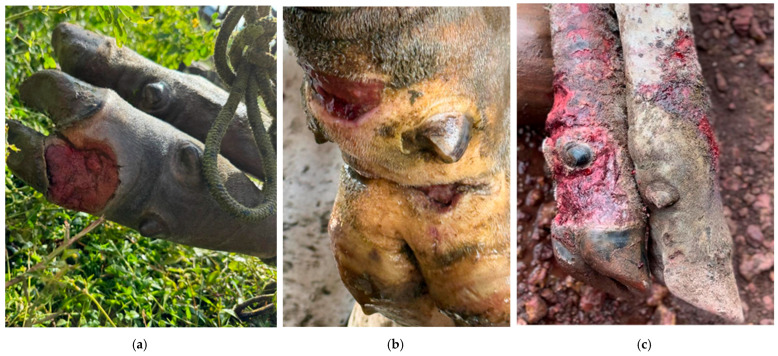
Representative cutaneous lesions observed in cattle affected by pythiosis. (**a**–**c**) Ulcerated, nodular, and exudative lesions affecting the distal limbs, with necrotic centers and irregular fibrotic borders.

Cutaneous lesions were consistent with those classically described for bovine pythiosis. Affected cattle presented multifocal nodular lesions that were ulcerated, hemorrhagic, and exudative, without visible kunkers, predominantly located on distal portions of the limbs, particularly in areas with frequent contact with water (Figure 4a–c). Lesions ranged from approximately 3 to 12 cm in diameter and commonly displayed necrotic centers surrounded by thickened, fibrotic margins. In approximately 15% of affected animals, lesions were associated with fistulous tracts draining serosanguinous exudate. Despite the extensive nature of some lesions, no evidence of deep musculoskeletal involvement was observed, and locomotion remained preserved throughout the clinical course.

### 3.3. Histopathological Findings

Histopathological evaluation of skin biopsies showed consistent lesions across all samples, characterized by marked dermal disorganization with fibroplasia, interlacing collagen bundles, moderate neovascularization, and a mild to moderate mononuclear inflammatory infiltrate compatible with granulation tissue. Multifocal superficial crusts composed of degenerated and intact neutrophils, amorphous basophilic and eosinophilic material, and occasional bacterial structures were observed, frequently replacing the epidermis. In sections with preserved epidermis, diffuse acanthosis with parakeratotic hyperkeratosis was noted. Occasional amorphous eosinophilic deposits surrounded by cellular debris were present; however, no consistent eosinophilic sleeves, angiocentric lesions, or morphologically recognizable oomycete hyphae were identified on hematoxylin and eosin or Grocott methenamine silver staining. Overall, the histopathological findings were nonspecific and did not allow definitive etiological characterization.

### 3.4. Molecular Diagnostic

Molecular analysis by quantitative real-time PCR (qPCR) targeting the internal transcribed spacer (ITS) region of *P. insidiosum* was performed samples collected from active lesion margins of 10 representative animals. All tested samples yielded positive amplification results. Amplification curves were comparable to those obtained for the positive control, with cycle threshold (Ct) values ranging from 19.4 to 25.6, consistent with a high pathogen DNA burden in the sampled lesions. No amplification was observed in negative controls, confirming assay specificity. These results provided definitive molecular confirmation of *P. insidiosum* as the etiological agent responsible for the outbreak. Representative amplification curves obtained from qPCR assays are provided as Supplementary Figure S1.

### 3.5. Therapeutic Management and Clinical Outcomes

Therapeutic management was implemented in a total of 213 animals presenting active, extensive, or progressive lesions requiring intervention, while the remaining affected cattle were managed conservatively due to mild or regressing lesions.

**Figure 5:**
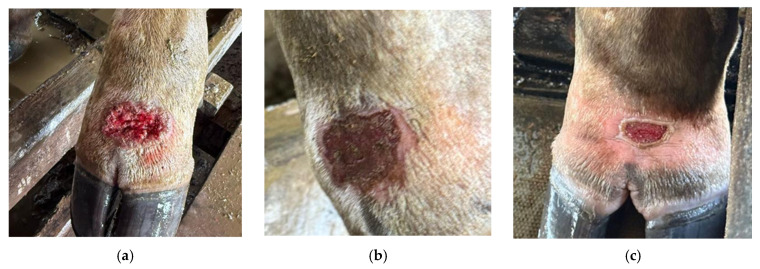
Sequential wound healing in a representative treated animal: (**a**) initial ulcerated lesion (Day 0); (**b**) marked reduction in exudation and lesion size (Day 15); (**c**) lesion in the process complete epithelialization and tissue repair (Day 30).

Two treatment protocols were applied based on lesion severity and extent. Protocol 1 consisted of intramuscular administration of triamcinolone acetonide (0.01 mg/kg) once weekly for two consecutive weeks, combined with topical application of 5% copper sulfate to the lesion surface once daily for 14 days. Protocol 2 consisted exclusively of topical application of 5% copper sulfate once daily until complete lesion closure. Among the 213 cattle treated under Protocol 1, 191 animals (89.7%) achieved complete clinical resolution, defined as full epithelialization, absence of exudation, and no lesion progression, within 21 to 35 days. The remaining 22 animals (10.3%) showed partial regression after the initial treatment course and required retreatment (Figure 5).

The 187 cattle managed under Protocol 2 showed complete clinical resolution within 30 to 40 days. Treatment duration, administration routes, and outcome definitions were standardized across all analyses to ensure internal consistency. All numerical data were reviewed and harmonized, and the sum of outcome categories corresponded exactly to the total number of treated animals reported in Table 1.

## 4. Discussion

This study documents the first molecularly confirmed outbreak of bovine pythiosis in the Amazon biome, which affected 400 cattle, and provides compelling evidence that *P. insidiosum* is no longer a sporadic or self-limiting condition in cattle raised in tropical flood-prone ecosystems [6,7,8,10]. Historically, bovine pythiosis has been described as an infrequent disease, usually affecting a small number of animals within a herd and often resolving spontaneously without major intervention [6,7,8,10]. In contrast, the magnitude, persistence, and clinical impact observed in the present outbreak indicate a marked shift in the epidemiological behavior of the pathogen, with important implications for animal health and livestock production systems in the Amazon region [6,7,8,25].

From an epidemiological perspective, the exceptionally high morbidity observed in this outbreak contrasts sharply with previous reports from southern, southeastern, and northeastern Brazil, where bovine pythiosis typically presents as isolated cases or small animal clusters [6,7,10,22]. The prolonged persistence of lesions and the need for pharmacological intervention in most affected cattle suggest that continuous exposure to flooded pastures played a decisive role in sustaining transmission [6,7,8]. In the Amazon biome, cattle management during the rainy season frequently involves prolonged grazing in waterlogged areas, limiting the feasibility of removing animals from contaminated environments. This scenario likely facilitated repeated exposure to infective zoospores, delaying spontaneous lesion regression and contributing to the chronicity observed in this outbreak [7,8].

The epidemiological scenario observed in the present outbreak was strongly shaped by environmental conditions known to favor the environmental amplification and host transmission of *P. insidiosum*. This aquatic oomycete exhibits an obligate association with warm, stagnant, or slow-moving freshwater systems, where it produces motile biflagellate zoospores capable of active chemotaxis and host invasion following prolonged cutaneous exposure [1,3,14]. In the Amazon biome, the convergence of sustained high temperatures, abundant organic substrates, and seasonal hydrological expansion creates highly permissive ecological niches for zoospore survival, dispersal, and host contact. Comparable eco-epidemiological contexts have been consistently implicated in livestock pythiosis outbreaks across tropical flood-prone regions and landscapes undergoing rapid environmental transformation [14,15,16,18]. In this investigation, the pronounced temporal aggregation of cases during the peak rainy season, coupled with the anatomical concentration of lesions in distal limb regions subjected to frequent water immersion, provides robust epidemiological evidence supporting a predominantly water-mediated transmission pathway.

At a broader scale, regional climatic dynamics appear to function as major upstream drivers of *P. insidiosum* epidemiology in the Amazon. The region is characterized by annual precipitation often exceeding 2000–3500 mm, high temperatures, resulting in recurrent and prolonged inundation of low-lying pastures and the establishment of semi-permanent aquatic microenvironments that sustain zoospore production and environmental persistence [15,16]. These hydrological conditions not only enhance pathogen maintenance in the environment but also substantially increase the duration and intensity of cattle exposure under extensive grazing systems. Importantly, climate projections for the Amazon indicate rising rainfall intensity and increased frequency of extreme precipitation events, trends that are expected to expand both the spatial footprint and seasonal window of pythiosis risk [15,16]. Collectively, these findings reinforce the conceptual framework that positions pythiosis as a climate-sensitive, water-associated disease whose emergence dynamics are tightly coupled to hydrometeorological variability and environmental change in tropical ecosystems.

Clinically, the lesions observed in this outbreak predominantly ulcerative, granulomatous, and affecting the distal limbs and ventral body regions were consistent with classical descriptions of bovine pythiosis [6,7,8]. However, lesion progression in the Amazon outbreak was notably more aggressive and prolonged, frequently requiring repeated therapeutic interventions. While histopathological findings such as pyogranulomatous dermatitis, eosinophilic infiltration, and poorly staining hyphal elements are traditionally considered supportive of pythiosis [7], histopathology alone proved insufficient for definitive diagnosis in this context. This reinforces previous concerns regarding the limited specificity of histopathology, particularly in chronic or atypical lesions, and highlights the critical importance of molecular confirmation [25,26].

Additionally, the inability to perform culture reflects both the intrinsic technical limitations of isolating *P. insidiosum* from clinical samples and the operational constraints of outbreak conditions, where molecular methods provide faster and more reliable diagnostic confirmation [9,10,12,13,25,26]. When successfully isolated, *P. insidiosum* typically grows on standard mycological media as sparsely septate hyaline hyphae, with zoospore production requiring specific aquatic induction conditions, which contributes to the low sensitivity and limited routine applicability of culture-based diagnosis [3,4].

In this outbreak, qPCR targeting the ITS region enabled rapid and accurate etiological confirmation of *P. insidiosum*, in line with previously validated molecular approaches [9,10,12,13,25,26]. Molecular diagnostics have consistently demonstrated superior sensitivity and specificity compared with conventional methods, particularly in early or chronic lesions and in situations where culture is unsuccessful [25,26]. However, access to molecular diagnostic infrastructure remains limited in many regions of the Amazon, underscoring a major gap in veterinary diagnostic capacity. Expanding access to PCR-based tools is therefore essential for improving surveillance, outbreak detection, and appropriate case management of emerging water-associated pathogens in tropical livestock systems.

The use of qPCR targeting the ITS region represents a critical advancement in the diagnosis of pythiosis, particularly in large-scale outbreak scenarios where rapid and reliable etiological confirmation is essential. Unlike histopathology, which may yield nonspecific findings, or culture, which is often unsuccessful for oomycetes, ITS-based qPCR provides high analytical sensitivity and specificity, enabling early detection even in lesions with low pathogen burden. Furthermore, this approach is particularly valuable in differentiating pythiosis from other ulcerative dermatoses of infectious or traumatic origin, which may present overlapping clinical features in cattle. In the context of tropical production systems, where diagnostic resources are often limited, the implementation of molecular tools such as qPCR can substantially improve outbreak response, surveillance, and disease management strategies [9,25,26,27].

Therapeutically, this study reports, for the first time, the field-scale use of intramuscular triamcinolone combined with topical copper sulfate for bovine pythiosis under outbreak conditions [28,29,30,31]. This combined approach resulted in clinical improvement and lesion regression in more than 80% of treated animals within 15–30 days. Although immunotherapy using *P. insidiosum* antigens has shown efficacy in individual cases and small series its cost, logistical complexity, and limited availability restrict its applicability at the herd level. In contrast, the corticosteroid-based protocol applied here appears to represent a pragmatic and scalable alternative, particularly in low-resource settings [19,29].

The observed therapeutic response is biologically plausible given the known pathophysiology of pythiosis, which is characterized by an exuberant Th2-polarized immune response, marked eosinophilic infiltration, and extensive tissue damage mediated by host inflammation rather than direct fungal invasion [4,15]. Corticosteroids may mitigate this dysregulated inflammatory response, while copper sulfate exerts local antimicrobial and astringent effects that may reduce pathogen burden and promote tissue repair [23]. Importantly, this study does not claim causal efficacy or superiority of this protocol but rather reports observational outcomes obtained during emergency outbreak management.

The observational design adopted in this study reflects the operational constraints of outbreak management under extensive cattle production systems in the Amazon biome. The implementation of controlled or randomized therapeutic trials was not feasible due to ethical obligations to provide immediate treatment to affected animals, the rapid progression of lesions, and the logistical challenges associated with managing large herds in flooded and geographically dispersed areas. These conditions precluded the use of untreated or placebo control groups. Future studies should consider prospective controlled or adaptive field trial designs in more accessible settings, enabling formal evaluation of therapeutic efficacy while maintaining animal welfare standards.

Despite the absence of significant body condition loss, consistent with previous observations in cattle [11,25], the economic impact of the outbreak was substantial. Direct treatment costs, labor, and prolonged healing times resulted in significant financial losses, particularly for smallholder farming systems. These findings emphasize that bovine pythiosis should no longer be regarded as a benign or negligible condition in tropical regions, but rather as a disease with tangible economic consequences. In addition, indirect losses related to reduced productivity, delayed weight gain, and increased management interventions may further amplify the economic burden of the disease [9,11,25,27].

Beyond animal health and production, the zoonotic potential of *P. insidiosum* merits consideration. Human pythiosis, though rare, has been increasingly reported in tropical regions, particularly among individuals with prolonged exposure to aquatic environments or underlying immunosuppression [5]. In this context, livestock outbreaks may serve as sentinel events, signaling environmental contamination and potential risk to human populations, especially in rural and riverine communities of the Amazon [30]. This highlights the importance of integrating veterinary findings into broader One Health surveillance frameworks.

Environmental management remains a critical but underutilized component of disease control. Restricting access to stagnant water bodies, implementing rotational grazing to avoid flooded pastures, and monitoring water quality parameters may reduce exposure risk. However, in ecosystems where flooding is unavoidable, proactive surveillance, early molecular diagnosis, and farmer education should be prioritized [25,27,31].

This study has important limitations, particularly the absence of environmental sampling, which precluded the direct identification of environmental reservoirs and transmission hotspots of *P. insidiosum*. Given the well-established association between this pathogen and aquatic ecosystems, the lack of water and soil analysis limits the ability to confirm local environmental persistence and quantify pathogen load in flooded pastures. Future investigations should incorporate standardized environmental DNA (eDNA) approaches, including qPCR-based detection targeting the ITS region [9,25,27].

The findings presented here indicate that *P. insidiosum* should be regarded not only as a neglected pathogen but also as an indicator of broader ecological and One Health vulnerabilities in tropical regions undergoing rapid environmental change [31,32,33]. The convergence of climate-driven hydrological instability, expanding livestock production, and limited diagnostic infrastructure in the Amazon biome [31] creates conditions conducive to the emergence and amplification of water-associated diseases such as pythiosis. Addressing these challenges will require integrated surveillance, improved molecular diagnostic capacity, and coordinated actions across animal, human, and environmental health sectors.

## 5. Conclusions

This study describes a large outbreak of cutaneous pythiosis in cattle from the Amazon biome, with etiological confirmation of *P. insidiosum* based on molecular detection under field conditions. The findings contribute descriptive epidemiological information for a region where bovine pythiosis remains poorly characterized and emphasize the importance of PCR-based methods for outbreak confirmation. All therapeutic interventions were implemented as part of routine outbreak management and were not designed to formally assess treatment efficacy. Accordingly, clinical outcomes should be interpreted strictly as observational findings. The study is limited by its observational design, restricted molecular sampling, absence of environmental investigation, and lack of long-term follow-up. Subsequent studies employing systematic epidemiological and controlled experimental approaches are warranted to further characterize transmission dynamics and optimize management strategies for pythiosis in tropical flood-prone environments.

## Figures and Tables

**Table 1:** Clinical outcomes of cattle affected by pythiosis according to therapeutic protocol: Protocol 1 (intramuscular triamcinolone combined with topical copper sulfate) and Protocol 2 (topical copper sulfate alone).

Outcome	Number of Animals	Retreatment	Recovered
Protocol 1	213	22	213
Protocol 2	187	0	187

## Data Availability

The original contributions presented in the study are included in the article; further inquiries can be directed to the corresponding author.

## Supplementary Materials

The following supporting information can be downloaded at: https://www.mdpi.com/article/10.3390/pathogens15040409/s1, Figure S1: Amplification plot of *Pythum insidiosus* detection in Real Time Polymerase Chain Reaction in bovine tissue. P.C: positive control; S1: bovine sample; N.C.: negative control.
