# Supplementary figures and images for: First Molecularly Confirmed Outbreak of Bovine Pythiosis Caused by Pythium insidiosum in the Amazon Biome
Source: Pathogens. 2026 Apr 9;15(4):409. doi: 10.3390/pathogens15040409 (PMC13118605; doi:10.3390/pathogens15040409)

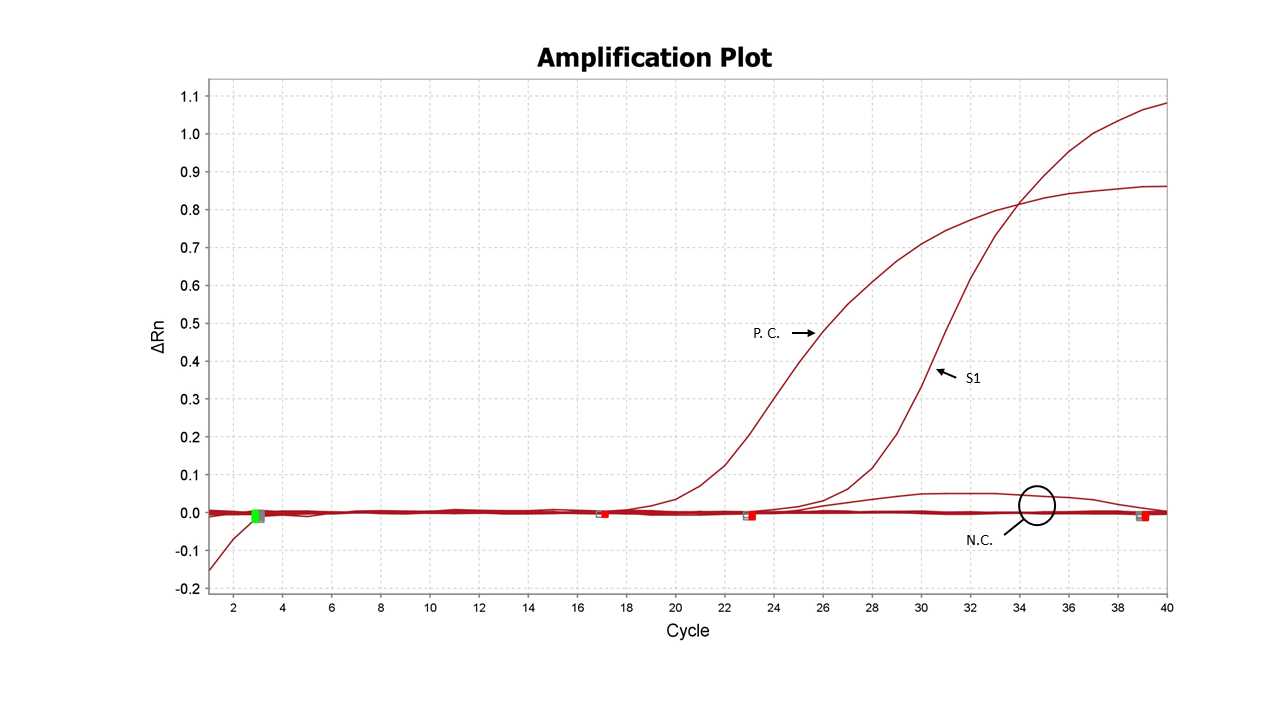

Supplement: Supplementary file 1 [file pathogens-15-00409-s001.zip › Figure S1.TIF]

## Slide 1
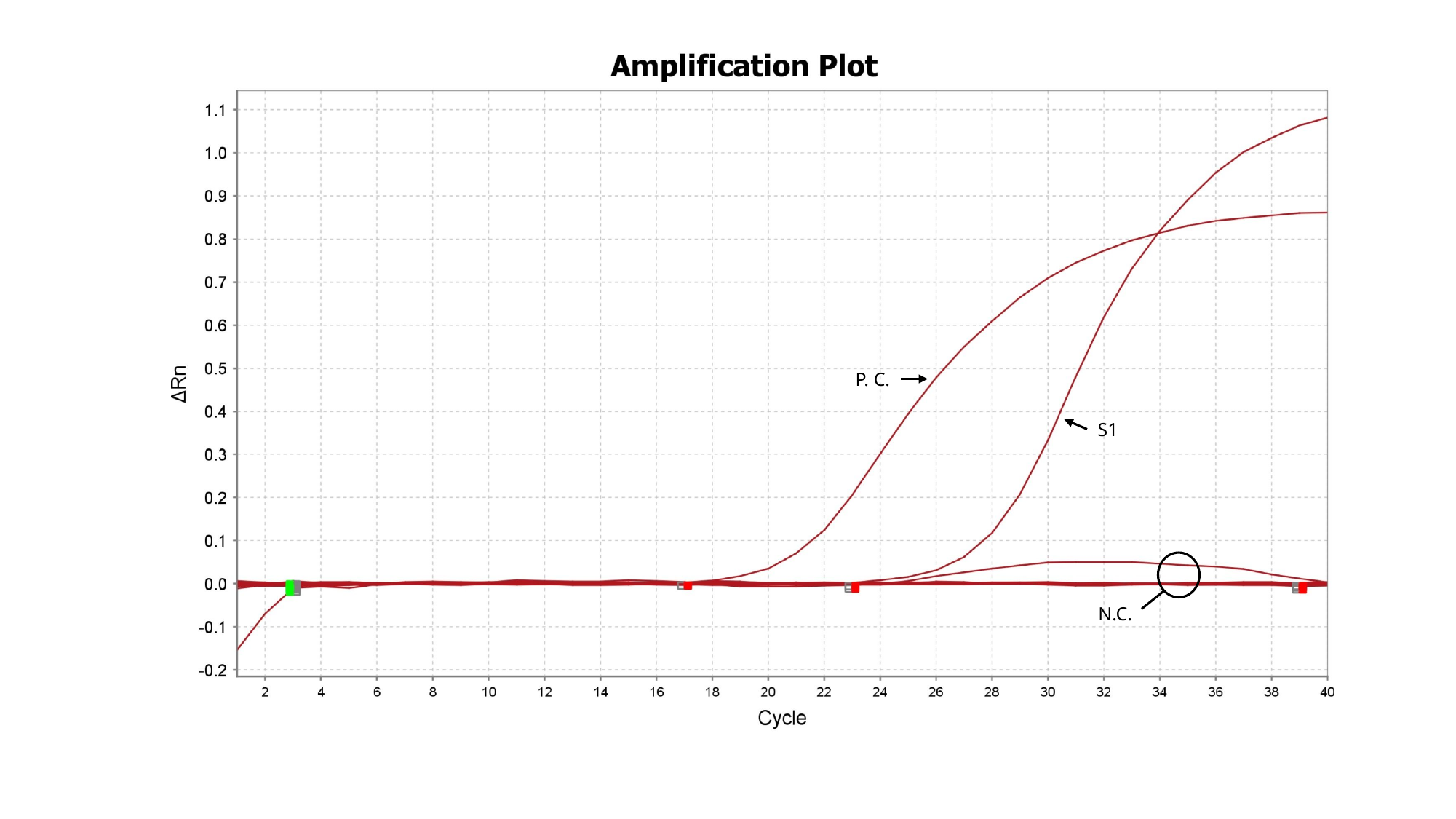

P. C.
S1
N.C.

Supplement: Supplementary file 1 [file pathogens-15-00409-s001.zip › Figure S1.pptx]
